# Tumor-associated macrophages correlate with the clinicopathological features and poor outcomes via inducing epithelial to mesenchymal transition in oral squamous cell carcinoma

**DOI:** 10.1186/s13046-015-0281-z

**Published:** 2016-01-15

**Authors:** Yong Hu, Meng-Ying He, Li-Fang Zhu, Cong-Chong Yang, Mei-Ling Zhou, Qiong Wang, Wei Zhang, Yang-Yu Zheng, Dong-Miao Wang, Zeng-Qi Xu, Yu-Nong Wu, Lai-Kui Liu

**Affiliations:** Jiangsu Key Laboratory of Oral Diseases, Nanjing Medical University, Nanjing, People’s Republic of China; Department of Basic Science of Stomatology, Affiliated Hospital of Stomatology, Nanjing Medical University, Postal#210029 136# Hanzhong Road, Nanjing, Jiangsu the People’s Republic of China; Department of Oral and Maxillofacial Surgery, Affiliated Hospital of Stomatology, Nanjing Medical University, Postal#210029 136# Hanzhong Road, Nanjing, Jiangsu the People’s Republic of China; Department of Stomatology, The First Affiliated Hospital of Soochow University, Suzhou, People’s Republic of China; Department of Stomatology, Suzhou Kowloon Hospital Shanghai Jiaotong University Medical School, Suzhou, People’s Republic of China

**Keywords:** Tumor-associated macrophages, CD68, CD163, Epithelial to mesenchymal transition, Oral squamous cell carcinoma

## Abstract

**Background:**

Both tumor-associated macrophages (TAMs) and the epithelial to mesenchymal transition (EMT) of cancer cells play key roles in promoting tumor progression. However, whether TAMs could induce EMT in the progression of oral squamous cell carcinoma (OSCC) remains undefined.

**Results:**

Here we detected the expression of macrophages markers CD68 and CD163, epithelial marker E-cadherin and mesenchymal marker vimentin in 127 OSCC patients by using semi-quantitative immunohistochemistry. CD68 and CD163 expression was not confined to the infiltrating TAMs, but also detected in cancer cells. The high number of CD68-positive macrophages was correlated with poor overall survival. Meanwhile, the expression of CD163 both in macrophages and in cancer cells was associated with poor overall survival and had a significant prognostic impact in OSCC. Importantly, the expression of CD163 in cancer cells had a significant relationship with E-cadherin and vimentin. Furthermore, the incubation of TAMs conditioned medium resulted in a fibroblast-like appearance of cancer cells (HN4, HN6 and SCC9) together with the decreased/increased expression of E-cadherin/ vimentin, which were correlated with the enhanced ability of migration and invasion.

**Conclusions:**

Our results indicate that TAMs could promote the EMT of cancer cells, thereby leading to the progression of oral cancer.

## Background

Oral squamous cell carcinoma (OSCC), the most common cancer in the head and neck, is associated with high metastasis and poor prognosis [1]. Despite advances in the surgical, chemotherapy and radiotherapy treatment option, the five-year survival rate has not been improved significantly in the past years.

Increasing studies indicate that the tumor microenvironment is important for cancer development and metastasis [2–4]. It contains diverse cells including leukocytes and fibroblasts. As one of major leukocytes, macrophages play a critical role in carcinogenesis and tumor progression [5]. They could be divided into two subgroups, the classically activated M1-type and the alternatively activated M2-type [6, 7]. The macrophages present in tumors are generally considered as tumor-associated macrophages (TAMs), which have a M2 phenotype and express CD68 and CD163 [7]. The high density of TAMs correlates with a poor prognosis and influences the initiation, progression and metastasis of various solid tumors [4, 8]. Recent studies demonstrate that the CD68-positive macrophages and CD163-positive macrophages correlate with histological grade and poor prognosis [1, 9–11]. Notably, cancer cells also express the macrophages antigen CD68 and CD163. The macrophage-like traits of cancer cells show a correlation to poor survival in malignant glioma, breast cancer, colorectal cancer, and malignant melanoma [12]. However, the mechanism underlying TAMs in OSCC invasiveness and metastasis has not been sufficiently analyzed. The precise role of macrophages antigen CD68 and CD163 in OSCC progression remains poorly understood.

Currently, the epithelial to mesenchymal transition (EMT) has been regarded to be involved in the progression of cancer. During the transition process, epithelial cells acquire fibroblastoid properties, and become motile and invasive. Importantly, our previous studies have found that the EMT contributes to invasion and metastasis in OSCC [13–15]. Recent studies demonstrate that cancer cells undergoing EMT at the invasive front of tumor tissue can establish a suitable microenvironment for tumor progression, where TAMs are always found [16, 17]. Accmulating evidences indicate that the TAMs can induce the cancer cells to undergo EMT and subsequently enhance the invasion and metastasis of breast cancer, cholangio carcinoma and hepatocellular carcinoma [16–19]. However, the interaction between the TAMs and cancer cells undergoing EMT in the OSCC progression remains unknown.

In the present study, we aim to investigate the incidence of CD68 and CD163 in relation to clinicopathological features and outcomes, then further insight into the interaction between TAMs and cancer cells undergoing EMT in OSCC progression. Explore the underlying mechanisms may identify innovative strategies to improve therapeutic efficacy of OSCC.

## Methods

### Patients and tissue specimens

One hundred twenty-seven primary OSCC specimens and 23 peritumoral tissue specimens were obtained from the Department of Oral Pathology and the Department of Oral Maxillofacial Surgery, Affiliated Hospital of Stomatology, Nanjing Medical University. The patients with primary OSCC had been treated with a wide excision surgery, with simultaneous an elective dissection of the regional lymph nodes or a classical radical neck dissection from August 2007 to April 2013 (74 men and 53 women; mean age, 61 years; rang, 34–88 years). None of the patients had received any preoperative radiation-chemotherapy. Peritumoral tissue samples were obtained from unaffected tissue surrounding the tumors. All the primary tumors were located in the tongue (*n* = 52), gingiva (*n* = 26), buccal mucosa (*n* = 31), lip (*n* = 4), floor of the mouth (*n* = 2), and others (*n* = 12). The tumor nest is the place at which tumor cells originates, accumulates, or develops, as the center on which a tumor forms. The tumor stroma is the microenvironment around the tumor cells. The TNM classification and clinical staging were carried according to the International Union Against Cancer (UICC). The pathological grade was classified into groups according to the WHO classification. The follow-up period ranged from 2 to 91 months (average: 41.2 months; median: 39 months). A total of 99 patients had at least a 2-year follow-up after treatment. This study protocol was approved according to the Ethics Committee of the Nanjing Medical University.

### Immunohistochemistry

The specimens were fixed in a 10 % formaldehyde solution and embedded in paraffin. Then, Paraffin tissue sections (4–5 μm) with xylene dewaxing and with graded alcohols rehydrating. Endogenous peroxidases were blocked for 10 min using 3 % hydrogen peroxide. Antigen retrieval with 0.01 M sodium citrate retrieval buffer (pH 6.0) was carried out by microwave heating for 20 min. The sections were then incubated overnight with antibodies for E-cadherin (Cell Signaling Technology, Danvers, MA, USA), vimentin (Santa Cruz Biotechnology, Santa Cruz, CA, USA), CD68 (Abcam, Cambridge, MA, USA), CD163 (Abcam) at 4 °C, and then incubated with appropriate secondary antibodies. Subsequently, the reaction product was visualized by incubation with 3,3’-diaminobenzidine and then counterstained with haematoxylin. Positive and negative controls were used in each staining run.

### Evaluation of immunoreactivity

The immunoreactivity of the E-cadherin, vimentin, CD68 and CD163 in cancer cells was evaluated using the same method which was described in our previous studies [13]. Immunoreactivity was semiquanitatively evaluated by using immunoreactive score. The final immunoreactivity was divided into negative (score = 0), low (1 ≤ score ≤ 4) and high (score > 4). CD68-positive and CD163-positive macrophages were quantified by two pathologists who were blinded to the clinical patient data. They detected each section at low magnification (100×) and identified the areas with the highest macrophage density. The number of macrophages was counted in ten random high-power fields (HPFs, 400×), the mean number of macrophages per HPF was evaluated after two counts. On the basis of the number of macrophages, the samples were divided into low and high groups by using cut-off values of 6/HPF in tumor nest and 8/HPF in tumor stroma.

### Cell cultures and preparation of TAMs

Human monocyte/macrophage cell line THP-1 was obtained from the Institute of Biochemistry and Cell Biology, Chinese Academy of Sciences, Shanghai, China. Human SCC9 was obtained from the American Type Culture Collection (ATCC, Manassas, VA, USA). Human HN4 and HN6 were kindly provided from the Shanghai Ninth People's Hospital, Shanghai, China. The HN4, HN6 and SCC9 cancer cells were maintained in a 1:1 mixture of Dulbecco’s modified Eagle’s medium and Ham’s F12 medium (Invitrogen, Burlington, Ontario, Canada) supplemented with 10 % fetal bovine serum (FBS, Invitrogen), 400 ng/mL hydrocortisone (Sigma-Aldrich, St Louis, MO, USA) and 1 % penicillin/streptomycin solution (Invitrogen). The THP-1 cells were maintained in RPMI‑1640 medium (Invitrogen) supplemented with above FBS and antibodies. To obtain TAMs cells, we treated the THP-1 cells with phorbolmyristate acetate (PMA, 10 ng/mL, Sigma, USA) for 48 h, and then added the macrophage colony-stimulating factor (M-CSF, 10 ng/mL Sigma, USA) into the medium for 24 h. After a thorough wash with PBS for two times, the adherent cells were cultured in RPMI‑1640 medium supplemented with FBS and antibodies. Five days later, the cells were collected and detected the expression of CD68 and CD163 (experimental procedure was detailed in immunofluorescence microscopy), and the conditioned medium (CM) from macrophages was harvested, clarified by centrifugation, used for mass spectral protein analysis (Invitrogen) and treating with the cancer cells. The HN4, HN6 and SCC9 cancer cells treating with CM were regarded as the experimental group (HN4-M, HN6-M and SCC9-M cells) for subsequent studies.

### Quantitative real-time PCR

The total RNA was extracted from cells by using the TRIzol reagent (Invitrogen), and mRNA was reverse transcribed into cDNA synthesis by using the 5 × PrimeScript RT Master Mix (TaKaRa, Otsu, Shiga, Japan) at 37 °C for 15 min and 85 °C for 5 s, according to the corresponding manufacturer’s protocol. Quantitative PCR (qPCR) was performed to evaluate the gene expression using 2 × SYBR Premix Ex Taq (TaKaRa) with a 7300 ABI Real-Time PCR System (Applied Biosystems, Foster City, CA, USA) following the conditions: 95 °C for 30 s, 95 °C for 5 s, and 60 °C for 31 s for 40 cycles. The relative mRNA levels were checked by the 2 (−ΔΔCT) method and were normalized to expression of GAPDH. GAPDH (5’-GAAGGTGAAGGTCGGAGTC-3’, 5’-GAGATGGTGATGGGATTTC-3’), E-cadherin (5’-TACACTGCCCAGGAGCCAGA-3’, 5’-TGGCACCAGTGTCCGGATTA-3’), vimentin (5’-TGAGTACCGGAGACAGGTGCAG-3’, 5’-TAGCAGCTTCAACGGCAAAGTTC-3’).

### Western blotting

Western blotting was performed as described in our previous study [14]. The blots were first probed with primary antibodies, such as anti-β-actin as control (1:1000, Santa Cruz Biotechnology), anti-E-cadherin (1:1000, Cell Signaling Technology), anti-vimentin (1:500, Santa Cruz Biotechnology), and then probed with appropriate secondary antibodies. The membranes were then developed by Immun-Star WesternC Kit (Bio-Rad, Hercules, CA, USA) products and bands were analyzed by exposure to film (Kodak, Japan).

### Immunofluorescence microscopy

Cells were grown on glass coverslips, fixed with 4 % paraformaldehyde (PFA) for 20 min at room temperature, permeabilised in 1 % Triton X-100 for 15 min, blocked with goat serum albumin for 30 min at 37 °C, then incubated with antibodies specific for CD68 (1:100, Abcam), CD163 (1:50, Abcam), E-cadherin (1:100, Cell Signaling Technology) and vimentin (1:100, Santa Cruz Biotechnology) followed by the appropriate secondary antibodies (1:50) and then further stained with DAPI (1:1000, Invitrogen) for 2 min. Cells were visualized using a Zeiss LSM-710 confocal microscope.

### Wound-healing and invasion assays

The cells were grown in 6-well plates to 90 % confluence and scraped with a pipette tip across the cell surface. After wounding, the suspension cells and debris were carefully removed, and the cells were incubated with serum-free medium. Migrating cells at the wound front were photographed after 24 h. Cells invasion assay was carried using Transwell filters (6.5-mm diameters, 8-μM pore sizes, Costar, Lowell, MA, USA). The filters were pre-coated for 30 min at 37 °C with Matrigel Basement Membrane Matrix (20 μL per square centimeter, BD Biosciences, MA, USA) mixed with serum free medium (1:3). Cells (4 × 10^5^) were washed with PBS, resuspended with 100 μL serum-free medium and added to the upper chamber, while a medium containing 10 % FBS was placed as a chemoattractant in the lower chamber. After incubating for 24 h at 37 °C in 5 % CO2, the chambers were fixed in 4 % PFA and stained with crystal violet (Sigma-Aldrich) for 30 min. The remaining cells and matrigel on the upper chamber were removed using cotton swabs, and the migratory cells present on the lower surface of the membrane were counted in ten random fields and photographed at 100x magnification (Olympus).

### Statistical analysis

The relationship between the CD68-positive macrophages, CD163-positive macrophages, CD68, CD163, E-cadherin, vimentin expression and clinicopathological parameters was analysed using the ANOVA, chi-square test, Fisher’s exact test, Cochran-Mantel-Haenszel chi-squared test. The associations between CD163, E-cadherin and vimentin expression were analysed using the Spearman rank correlation analysis. The overall survival rate was estimated using the Kaplan–Meier method and compared by the log-rank test. The prognostic analysis was carried out with univariate Cox regression models. All the data were analyzed using SPSS statistical software (for Windows, version 16.0). *P*-value less than 0.05 was considered statistically significant.

## Results

### CD68-positive macrophages and CD163-positive macrophages in oral squamous cell carcinoma and peritumoral tissue

**Fig. 1:**
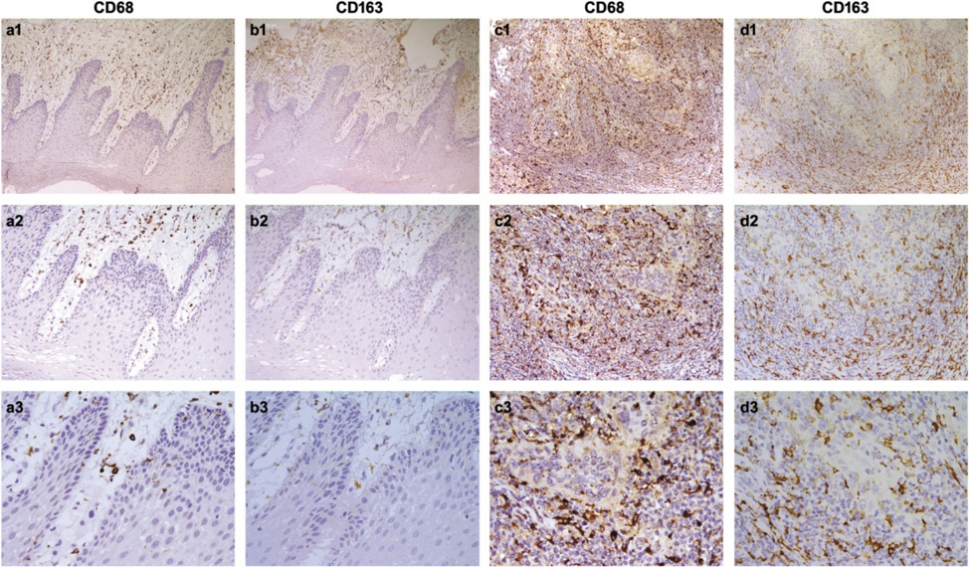
CD68-positive macrophages and CD163-positive macrophages were examined by immunohistochemistry. CD68 staining **a**1, **a**2, **a**3 and negative staining of CD163 was found in peritumoral stroma (**b**1, **b**2, **b**3). CD68-positive macrophages (**c**1, **c**2, **c**3) and CD163-positive macrophages (**d**1, **d**2, **d**3) were found both in tumor nest and stroma. (**a**1-**d**1, 100×; **a**2-**d**2, 200×; **a**3-**d**3, 400× )

In this study, we assessed the expression of two TAMs markers CD68 and CD163 in tumor or peritumoral tissue specimens. Of note, CD68-positive macrophages were detected only in peritumoral stroma (Fig. 1 a1, a2, a3), where had no CD163-positive macrophages (Fig. 1 b1, b2, b3). However, CD68 and CD163 were detected on the plasma membrane or in the cytoplasm of the macrophages both in tumor nest and stroma (Fig. 1 c1, c2, c3; 1d1, d2, d3).

The number of CD68-positive macrophages in the nest and stroma of tumor was increased in 59 % (75 out of 127) and 66 % (84 out of 127) of samples, respectively. Interestingly, the increase of CD68-positive macrophages in the nest but not stroma correlates with the tumor location (*P* = 0.034) and mortality (*P* = 0.005, Table 1). In contrast, the density of CD163-positive macrophages in the tumor nest and stroma was higher in 59 % (75 out of 127) and 57 % (73 out of 127) of total samples (Table 2). Importantly, the density of CD163-positive macrophages in tumor nest was significantly associated with the sex (*P* = 0.037) and mortality (*P* = 0.044), while that in the tumor stroma was significantly correlated with the recurrence (*P* = 0.008) and mortality (*P* = 0.04). In addition, we analyzed the expression of CD68 and CD163 in 23 peritumoral tissue samples (Table 3). 52 % (12 out of 23, *P* = 0.374) of samples showed relatively high density of CD68-positive macrophages in peritumoral stroma, however, the density of CD163-positive macrophages (*P* < 0.001) was none in all samples. Based on these results, we conclude that the infiltration CD163-positive macrophages have a prognostic significance.

**Table 1:** Correlation between clinical parameters and CD68+ macrophages counts in tumor nest and tumor stroma

Variable	No.	CD68+ macrophages counts in tumor nest			CD68+ macrophages counts in tumor stroma		
		Low(≤6/HPF)	High(>6/HPF)	*P*	Low(≤8/HPF)	High(>8/HPF)	*P*
Sex				**0.176**			**0.060**
Male	**74**	**34**	**40**		**30**	**44**	
Female	**53**	**18**	**35**		**13**	**40**	
Age (years)				**0.773**			**0.927**
≤50	**26**	**10**	**16**		**9**	**17**	
>50	**101**	**42**	**59**		**34**	**67**	
Tumor location				**0.034**			**0.346**
Tongue	**52**	**29**	**23**		**20**	**32**	
Gingiva	**26**	**7**	**19**		**8**	**18**	
Buccal mucosa	**31**	**8**	**23**		**6**	**25**	
Lip	**4**	**3**	**1**		**2**	**2**	
Floor of the mouth	**2**	**1**	**1**		**1**	**1**	
Others	**12**	**4**	**8**		**6**	**6**	
Tumor size				**0.665**			**0.369**
T1	**21**	**9**	**12**		**7**	**14**	
T2	**68**	**28**	**40**		**21**	**47**	
T3	**26**	**12**	**14**		**8**	**18**	
T4	**12**	**3**	**9**		**7**	**5**	
Lymph node metastasis				**0.850**			**0.321**
N0	**72**	**30**	**42**		**27**	**45**	
N (+)	**55**	**22**	**33**		**16**	**39**	
Metastasis				**0.793**			**0.627**
M0	**125**	**51**	**74**		**42**	**83**	
M (+)	**2**	**1**	**1**		**1**	**1**	
Clinical stage				**0.262**			**0.699**
I	**12**	**5**	**7**		**4**	**8**	
II	**42**	**19**	**23**		**17**	**25**	
III	**34**	**16**	**18**		**8**	**26**	
IV	**39**	**12**	**27**		**14**	**25**	
Pathological grade				**0.135**			**0.296**
I	**76**	**34**	**42**		**24**	**52**	
II	**42**	**18**	**24**		**13**	**29**	
III	**9**	**0**	**9**		**6**	**3**	
Recurrence				**0.111**			**0.259**
No	**83**	**39**	**44**		**25**	**58**	
Yes	**42**	**13**	**29**		**18**	**24**	
Lost	**2**	**0**	**2**		**0**	**2**	
Follow-up				**0.005**			**0.218**
Live	**91**	**45**	**46**		**27**	**64**	
Dead	**33**	**7**	**26**		**15**	**18**	
Lost	**3**	**0**	**3**		**1**	**2**	

No., number of patients; HPF, high-power field. N0, no lymph node metastasis; N (+), node metastasis. M0, no metastasis; M(+), metastasis

Bold values signify *P* < 0.05

**Table 2:** Correlation between clinical parameters and CD163^+^ macrophages counts in tumor nest and tumor stroma

Variable	No.	CD163+ macrophages counts in tumor nest			CD163+ macrophages counts in tumor stroma		
		Low(≤6/HPF)	High(>6/HPF)	*P*	Low(≤8/HPF)	High(>8/HPF)	*P*
Sex				**0.037**			**0.846**
Male	**74**	**36**	**38**		**32**	**42**	
Female	**53**	**16**	**37**		**22**	**31**	
Age (years)				**0.874**			**0.674**
≤50	**26**	**11**	**15**		**12**	**14**	
>50	**101**	**41**	**60**		**42**	**59**	
Tumor location				**0.389**			**0.057**
Tongue	**52**	**24**	**28**		**22**	**30**	
Gingiva	**26**	**9**	**17**		**17**	**9**	
Buccal mucosa	**31**	**9**	**22**		**7**	**24**	
Lip	**4**	**3**	**1**		**2**	**2**	
Floor of the mouth	**2**	**1**	**1**		**1**	**1**	
Others	**12**	**6**	**6**		**5**	**7**	
Tumor size				**0.921**			**0.335**
T1	**21**	**7**	**14**		**7**	**14**	
T2	**68**	**31**	**37**		**30**	**38**	
T3	**26**	**8**	**18**		**9**	**17**	
T4	**12**	**6**	**6**		**8**	**4**	
Lymph node metastasis				**0.200**			**0.889**
N0	**72**	**33**	**39**		**31**	**41**	
N (+)	**55**	**19**	**36**		**23**	**32**	
Metastasis				**0.235**			**0.829**
M0	**125**	**52**	**73**		**53**	**72**	
M (+)	**2**	**0**	**2**		**1**	**1**	
Clinical stage				**0.059**			**0.725**
I	**12**	**5**	**7**		**4**	**8**	
II	**42**	**21**	**21**		**21**	**21**	
III	**34**	**16**	**18**		**13**	**21**	
IV	**39**	**10**	**29**		**16**	**23**	
Pathological grade				**0.211**			**0.469**
I	**76**	**35**	**41**		**35**	**41**	
II	**42**	**13**	**29**		**14**	**28**	
III	**9**	**4**	**5**		**5**	**4**	
Recurrence				**0.22**			**0.01**
No	**83**	**38**	**45**		**28**	**55**	
Yes	**42**	**13**	**29**		**24**	**18**	
Lost	**2**	**1**	**1**		**2**	**0**	
Follow-up				**0.04**			**0.040**
Live	**91**	**43**	**48**		**34**	**57**	
Dead	**33**	**8**	**25**		**17**	**16**	
Lost	**3**	**1**	**2**		**3**	**0**	

No., number of patients; HPF, high-power field. N0, no lymph node metastasis; N (+), node metastasis. M0, no metastasis; M(+), metastasis

Bold values signify *P* < 0.05

**Table 3:** Relationship between CD68+, CD163+ macrophages counts in tumor nest and peritumoral epithelia

Variable	No.	CD68+ macrophages counts			CD163+ macrophages counts		
		High(>6/HPF)	Low(≤6/HPF)	*P*	High(>6/HPF)	Low(≤6/HPF)	*P*
				**0.000**			**0.000**
Normal	**23**	**0**	**23**		**0**	**23**	
Tumor	**127**	**75**	**52**		**75**	**52**	
Relationship between CD68+, CD163+ macrophages counts in tumor stroma and peritumoral stroma							
Variable	No.	CD68+ macrophages counts			CD163+ macrophages counts		
		High(>8/HPF)	Low(≤8/HPF)	*P*	High(>8/HPF)	Low(≤8/HPF)	*P*
				**0.374**			**0.000**
Normal	**23**	**12**	**11**		**0**	**23**	
Tumor	**127**	**84**	**43**		**73**	**54**	

No., number of patients; HPF, high-power field

Bold values signify *P* < 0.05

### Expression of CD68 and CD163 in oral squamous cell carcinoma patients

**Fig. 2:**
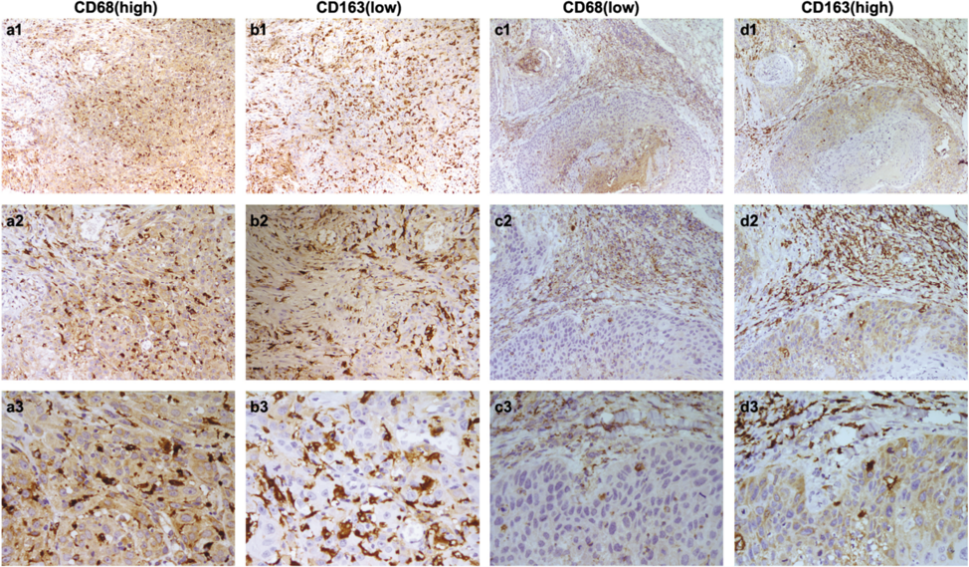
CD68 and CD163 expression was examined in tumor cells by immunohistochemistry. High expression of CD68 **a**1, **a**2, **a**3 and low expression of CD163 (**b**1, **b**2, **b**3) was found in tumor cells. Low expression of CD68 (**c**1, **c**2, **c**3) and high expression of CD163 (**d**1, **d**2, **d**3) was found in tumor cells. (**a**1-**d**1, 100×; **a**2-**d**2, 200×; **a**3-**d**3, 400×)

In addition to TAMs, tumor cells also expressed CD68 (Fig. 2 a1, a2, a3; 2c1, c2, c3) and CD163 (Fig. 2 b1, b2, b3;2 d1, d2, d3) on the plasma membrane and in the cytoplasm as detected by the immunohistochemical staining. CD68-positive tumor cells were detected in 83 % of samples, of which 79 cases (Fig. 2 a1, a2, a3) and 27 cases (Fig. 2 c1, c2, c3) showed relatively high and low expression of CD68. In contrast, CD163-positive tumor cells were found in 62 % of samples, of which 36 cases (Fig. 2 d1, d2, d3) and 43 cases (Fig. 2 b1, b2, b3) have increased and decreased expression of CD163. The expression levels of CD68 in tumor cells were only associated with lymph node metastasis (*P* = 0.044, Table 4), while the expression of CD163 was significantly associated with the recurrence (*P* = 0.013) and mortality (*P* = 0.001). This is the first time showed that a fraction of OSCC tumor cells exhibited definite CD68 and CD163 expression. More important, the high expression of CD163 was correlated with poor survival.

**Table 4:** Relationship between CD68, CD163 expression levels of tumors and clinical variables

Variable	No.	CD68				CD163			
		N	L	H	P	N	L	H	P
Sex					**0.081**				**0.008**
Male	**74**	**15**	**19**	**40**		**35**	**25**	**14**	
Female	**53**	**6**	**8**	**39**		**13**	**18**	**22**	
Age (years)					**0.668**				**0.642**
≤50	**26**	**5**	**4**	**17**		**8**	**9**	**9**	
>50	**101**	**16**	**23**	**62**		**40**	**34**	**27**	
Tumor location					**0.324**				**0.405**
Tongue	**52**	**10**	**12**	**30**		**20**	**17**	**15**	
Gingiva	**26**	**3**	**2**	**21**		**8**	**11**	**7**	
Buccal mucosa	**31**	**3**	**8**	**20**		**11**	**9**	**11**	
Lip	**4**	**2**	**1**	**1**		**4**	**0**	**0**	
Floor of the mouth	**2**	**0**	**1**	**1**		**0**	**1**	**1**	
Others	**12**	**3**	**3**	**6**		**5**	**5**	**2**	
Tumor size					**0.419**				**0.124**
T1	**21**	**5**	**3**	**13**		**9**	**4**	**8**	
T2	**68**	**9**	**14**	**45**		**23**	**24**	**21**	
T3	**26**	**4**	**5**	**17**		**14**	**7**	**5**	
T4	**12**	**3**	**5**	**4**		**2**	**8**	**2**	
Lymph node metastasis					**0.044**				**0.098**
N0	**72**	**15**	**19**	**38**		**33**	**22**	**17**	
N (+)	**55**	**6**	**8**	**41**		**15**	**21**	**19**	
Metastasis					**0.542**				**0.535**
M0	**125**	**21**	**27**	**77**		**48**	**42**	**35**	
M (+)	**2**	**0**	**0**	**2**		**0**	**1**	**1**	
Clinical stage					**0.564**				**0.114**
I	**12**	**4**	**2**	**6**		**7**	**2**	**3**	
II	**42**	**7**	**10**	**25**		**17**	**14**	**11**	
III	**34**	**4**	**7**	**23**		**14**	**9**	**11**	
IV	**39**	**6**	**8**	**25**		**10**	**18**	**11**	
Pathological grade					**0.986**				**0.537**
I	**76**	**12**	**17**	**47**		**31**	**25**	**20**	
II	**42**	**8**	**6**	**28**		**15**	**16**	**11**	
III	**9**	**1**	**4**	**4**		**2**	**2**	**5**	
Recurrence					**0.870**				**0.01**
No	**83**	**14**	**16**	**53**		**35**	**32**	**16**	
Yes	**42**	**7**	**11**	**24**		**13**	**10**	**19**	
Lost	**2**	**0**	**0**	**2**		**0**	**1**	**1**	
Follow-up					**0.560**				**0**
Live	**91**	**16**	**17**	**58**		**41**	**33**	**17**	
Dead	**33**	**5**	**10**	**18**		**7**	**9**	**17**	
Lost	**3**	**0**	**0**	**3**		**0**	**1**	**2**	

No., number of patients; N, negative; L low expression; H, high expression. N0, no lymph node metastasis; N (+), node metastasis. M0, no metastasis; M(+), metastasis

Bold values signify *P* < 0.05

### Expression of E-cadherin and vimentin in oral squamous cell carcinoma patients

**Fig. 3:**
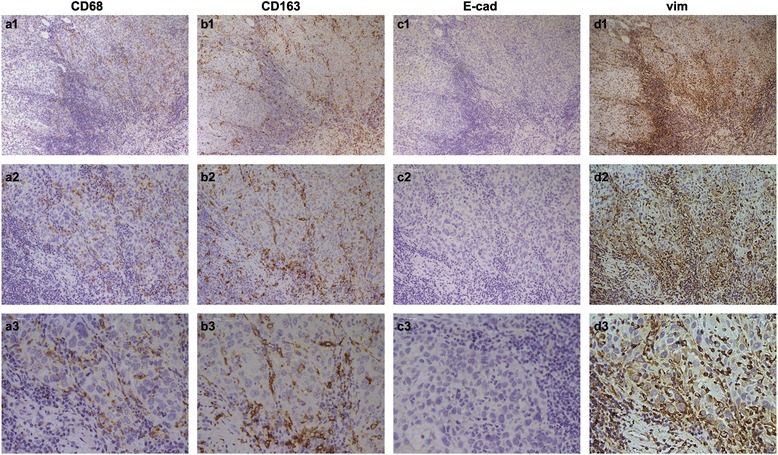
CD68-positive macrophages, CD163-positive macrophages, E-cadherin and vimentin was examined by immunohistochemistry. In oral squamous cell carcinoma patients, the absence or reduced expression of CD68-positive macrophages **a**1, **a**2, **a**3 and increased expression of CD163-positive macrophages (**b**1, **b**2, **b**3) was observed in tumor tissue, where have no expression of E-cadherin (**c**1, **c**2, **c**3), but strong cytoplasmic staining of vimentin (**d**1, **d**2, **d**3). (**a**1-**d**1, 100×; **a**2-**d**2, 200×; **a**3-**d**3, 400×)

**Fig. 4:**
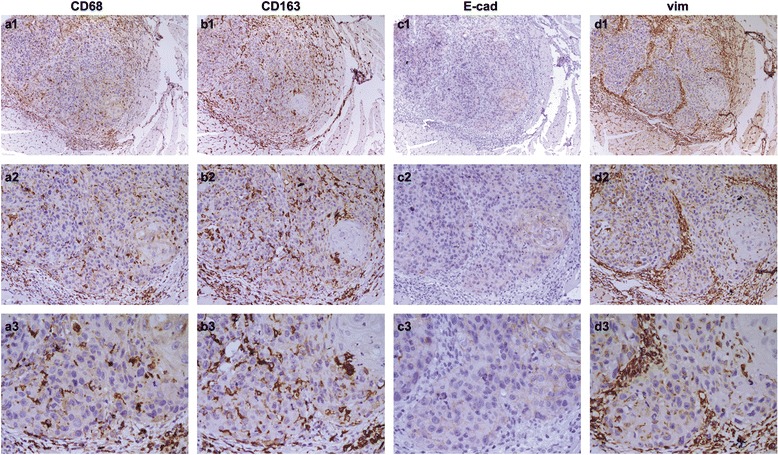
Expression of CD68, CD163, E-cadherin and vimentin in tumor cells. Increased expression of CD68 **a**1, **a**2, **a**3 and CD163 (**b**1, **b**2, **b**3) was observed in tumor cells, whereas there was absence or reduced expression of E-cadherin (**c**1, **c**2, **c**3), but strong cytoplasmic expression of vimentin (**d**1, **d**2, **d**3) at the invasive front of oral squamous cell carcinoma (**a**1-**d**1, 100×; **a**2-**d**2, 200×; **a**3-**d**3, 400×)

It has been reported that the number of TAMs is associated with EMT in tumor samples [16]. So we are wondering whether this relationship also exists in OSCC. Therefore, E-cadherin and vimentin, markers of EMT were detected by IHC. As shown in Fig. 3, the absence or reduced expression of CD68-positive macrophages (Fig. 3 a1, a2, a3) and increased expression of CD163-positive macrophages (Fig. 3 b1, b2, b3) was observed in tumor tissue, where have no expression of E-cadherin (Fig. 3 c1, c2, c3), but strong cytoplasmic staining of vimentin (Fig. 3 d1, d2, d3). Otherwise, Increased expression of CD68 (Fig. 4 a1, a2, a3) and CD163 (Fig. 4 b1, b2, b3) was also observed in tumor cells, whereas there was absence or reduced expression of E-cadherin (Fig. 4 c1, c2, c3), but strong cytoplasmic expression of vimentin (Fig. 4 d1, d2, d3) at the invasive front of the tumor. In the cancerous tissue, 99 out of 127 tumors express E-cadherin (78 %), the low expression, 36 cases; the high expression, 63 cases; and the negative expression, 28 (22 %) cases. Then, we check the expression levels of the tumors invasive front, 92 out of 127 (72 %) exhibit E-cadherin expression, the low expression, 52 cases; the high expression, 40 cases; and the negative expression, 35 (28 %) cases. As shown in Table 5 and Table 6, there was no significant relationship between the expression of E-cadherin and clinical variables, including sex, age, tumor location, tumor size, lymph node, metastasis, clinical stage, pathological grade, recurrence and mortality. Meanwhile, of the 127 tumors, 71 (56 %) cases express vimentin, the low expression, 39 cases; the high expression, 32 cases; and the negative expression, 56 (44 %) cases. Besides, high cytoplasmic staining of vimentin was also observed at the invasive front of tumors in 35 % (45 out of 127) patients. Despite no significant correlation with sex, age, and location and size of tumor, the expression of vimentin was associated with the metastasis into lymph nodes, clinical stage, pathological grade, recurrence and mortality. Especially at the tumor invasion front, the increased expression of vimentin was observed in 55 % (23 out of 42) patients with recurrence, and in 61 % (20 out of 33) patients with poor survival.

**Table 5:** Relationship between E-cadherin, vimentin expression levels of tumors and clinical variables

Variable	No.	E-cadherin				Vimentin			
		N	L	H	P	N	L	H	P
Sex					**0.990**				**0.205**
Male	**74**	**16**	**21**	**37**		**37**	**22**	**15**	
Female	**53**	**12**	**15**	**26**		**19**	**17**	**17**	
Age (years)					**0.257**				**0.383**
≤50	**26**	**7**	**4**	**15**		**12**	**10**	**4**	
>50	**101**	**21**	**32**	**48**		**44**	**29**	**28**	
Tumor location					**0.444**				**0.345**
Tongue	**52**	**13**	**12**	**27**		**26**	**16**	**10**	
Gingiva	**26**	**3**	**6**	**17**		**12**	**10**	**4**	
Buccal mucosa	**31**	**9**	**11**	**11**		**10**	**8**	**13**	
Lip	**4**	**1**	**1**	**2**		**1**	**2**	**1**	
Floor of the mouth	**2**	**1**	**1**	**0**		**2**	**0**	**0**	
Others	**12**	**1**	**5**	**6**		**5**	**3**	**4**	
Tumor size					**0.848**				**0.819**
T1	**21**	**3**	**7**	**11**		**10**	**6**	**5**	
T2	**68**	**18**	**19**	**31**		**27**	**24**	**17**	
T3	**26**	**5**	**7**	**14**		**12**	**5**	**9**	
T4	**12**	**2**	**3**	**7**		**7**	**4**	**1**	
Lymph node metastasis					**0.150**				**0.010**
N0	**72**	**20**	**17**	**35**		**40**	**19**	**13**	
N (+)	**55**	**8**	**19**	**28**		**16**	**20**	**19**	
Metastasis					**0.678**				**0.050**
M0	**125**	**28**	**35**	**62**		**56**	**39**	**30**	
M (+)	**2**	**0**	**1**	**1**		**0**	**0**	**2**	
Clinical stage					**0.071**				**0.046**
I	**12**	**3**	**1**	**8**		**9**	**2**	**1**	
II	**42**	**15**	**10**	**17**		**21**	**12**	**9**	
III	**34**	**4**	**12**	**18**		**13**	**10**	**11**	
IV	**39**	**6**	**13**	**20**		**13**	**15**	**11**	
Pathological grade					**0.441**				**0.001**
I	**76**	**16**	**19**	**41**		**40**	**25**	**11**	
II	**42**	**11**	**13**	**18**		**15**	**13**	**14**	
III	**9**	**1**	**4**	**4**		**1**	**1**	**7**	
Recurrence					**0.922**				**0.05**
No	**83**	**18**	**24**	**41**		**43**	**24**	**16**	
Yes	**42**	**9**	**12**	**21**		**13**	**14**	**15**	
Lost	**2**	**1**	**0**	**1**		**0**	**1**	**1**	
Follow-up					**0.747**				**0.014**
Live	**91**	**18**	**27**	**46**		**47**	**27**	**17**	
Dead	**33**	**9**	**9**	**15**		**9**	**11**	**13**	
Lost	**3**	**1**	**0**	**2**		**0**	**1**	**2**	

No., number of patients; N, negative; L low expression; H, high expression; N0, no nodal metastasis; N(+), nodal metastasis. M0, no metastasis; M(+), metastasis

Bold values signify *P* < 0.05

**Table 6:** Relationship between E-cadherin, vimentin expression levels of the tumors invasive front and clinical variables

Variable	No.	E-cadherin				Vimentin			
		N	L	H	P	N	L	H	P
Sex					**0.554**				**0.024**
Male	**74**	**20**	**28**	**26**		**37**	**18**	**19**	
Female	**53**	**15**	**24**	**14**		**19**	**8**	**26**	
Age (years)					**0.988**				**0.209**
≤50	**26**	**7**	**11**	**8**		**12**	**8**	**6**	
>50	**101**	**28**	**41**	**32**		**44**	**18**	**39**	
Tumor location					**0.123**				**0.508**
Tongue	**52**	**15**	**21**	**16**		**26**	**9**	**17**	
Gingiva	**26**	**6**	**6**	**14**		**12**	**7**	**7**	
Buccal mucosa	**31**	**11**	**15**	**5**		**10**	**6**	**15**	
Lip	**4**	**1**	**1**	**2**		**1**	**2**	**1**	
Floor of the mouth	**2**	**1**	**1**	**0**		**2**	**0**	**0**	
Others	**12**	**1**	**8**	**3**		**5**	**2**	**5**	
Tumor size					**0.970**				**0.818**
T1	**21**	**5**	**8**	**8**		**10**	**4**	**7**	
T2	**68**	**21**	**28**	**19**		**27**	**16**	**25**	
T3	**26**	**6**	**13**	**7**		**12**	**4**	**10**	
T4	**12**	**3**	**3**	**6**		**7**	**2**	**3**	
Lymph node metastasis					**0.236**				**0.003**
N0	**72**	**23**	**25**	**24**		**40**	**15**	**17**	
N (+)	**55**	**12**	**27**	**16**		**16**	**11**	**28**	
Metastasis					**0.665**				**0.159**
M0	**125**	**35**	**51**	**39**		**56**	**26**	**43**	
M (+)	**2**	**0**	**1**	**1**		**0**	**0**	**2**	
Clinical stage					**0.236**				**0.043**
I	**12**	**3**	**3**	**6**		**9**	**2**	**1**	
II	**42**	**17**	**13**	**12**		**21**	**8**	**13**	
III	**34**	**5**	**19**	**10**		**13**	**6**	**15**	
IV	**39**	**10**	**17**	**12**		**13**	**10**	**16**	
Pathological grade					**0.659**				**0.007**
I	**76**	**20**	**29**	**27**		**40**	**16**	**20**	
II	**42**	**13**	**20**	**9**		**15**	**10**	**17**	
III	**9**	**2**	**3**	**4**		**1**	**0**	**8**	
Recurrence					**0.460**				**0.01**
No	**83**	**22**	**32**	**29**		**43**	**19**	**21**	
Yes	**42**	**12**	**20**	**10**		**13**	**6**	**23**	
Lost	**2**	**1**	**0**	**1**		**0**	**1**	**1**	
Follow-up					**0.301**				**0.001**
Live	**91**	**22**	**39**	**30**		**47**	**21**	**23**	
Dead	**33**	**12**	**13**	**8**		**9**	**4**	**20**	
Lost	**3**	**1**	**0**	**2**		**0**	**1**	**2**	

No., number of patients; N, negative; L low expression; H, high expression. N0, no nodal metastasis; N(+), nodal metastasis. M0, no metastasis; M(+), metastasis

Bold values signify *P* < 0.05

### Association between the expression of CD163, E-cadherin and vimentin

To investigate the role of TAMs in tumor progression, the relationship between the CD163, E-cadherin and vimentin was analyzed. As shown in Table 7, the levels of CD163 expression in tumor cells were positively associated with the expression of E-cadherin (*P* = 0.04 and *P* = 0.038, respectively) and vimentin (*P* < 0.001 and *P* < 0.001, respectively) in tumors and at the tumor invasion front. However, the CD163-positive macrophages have no association with E-cadherin and vimentin (data not shown).

**Table 7:** Relationship between CD163, E-cadherin and vimentin

Variable	CD163
	***P***
**E-cadherin**	
In tumor	**0.040**
At tumor invasive front	**0.038**
**Vimentin**	
In tumor	**0.000**
At tumor invasive front	**0.000**

Bold values signify *P* < 0.05

### Survival analysis

**Fig. 5:**
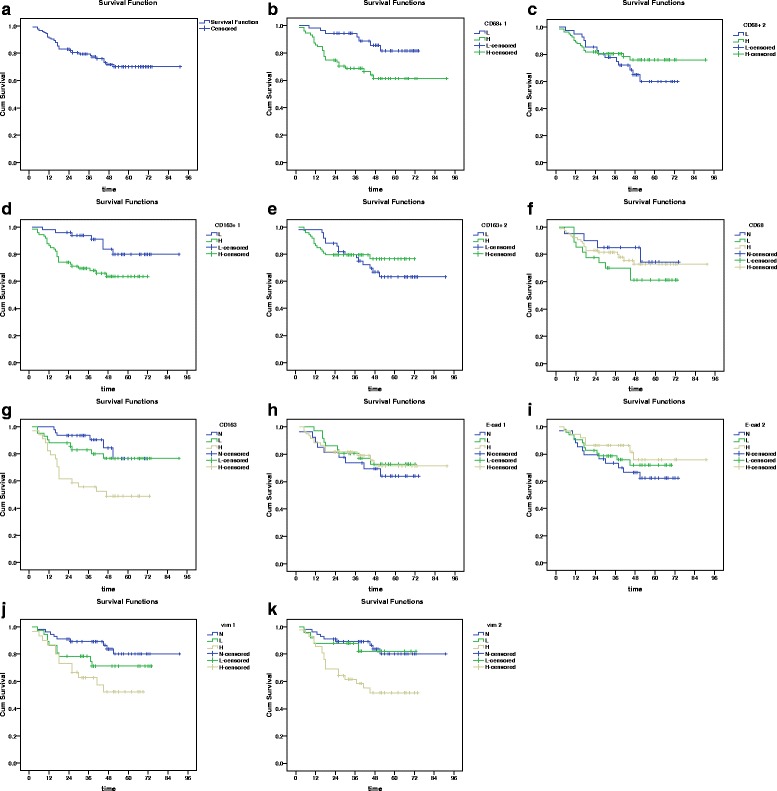
Survival cures of 127 oral squamous cell carcinoma patients. **a** Overall survival (OS) curves of 127 oral squamous cell carcinoma patients. In OSCC patients, the expression of CD68-positive macrophages and CD163-positive macrophages in tumor nest, CD163 in tumor cells, vimentin both in tumor and at the tumor invasion front was associated with poor OS (**b**, **d**, **g**, **j**, **k**). The expression of CD68-positive macrophages and CD163-positive macrophages in tumor stroma, CD68 in tumor cells, E-cadherin both in tumor and at the tumor invasion front was not associated with poor OS (**c**, **e**, **f**, **h**, **i**). L, low (≤ 6/ high-power field); H, high (≥ 8/ high-power field); N, negative; L, low expression; H, high expression. CD68+ 1: CD68-positive macrophages counts in tumor nest; CD68+ 2: CD68-positive macrophages counts in tumor stroma; CD163+ 1: CD163-positive macrophages counts in tumor nest; CD163+ 2: CD163-positive macrophages counts in tumor stroma; CD68: CD68 expression in tumors; CD163: CD163 expression in tumors; E-cad 1: E-cadherin expression in tumor; E-cad 2: E-cadherin expression at the invasive front of tumor; vim 1: vimentin expression in tumor; vim 2: vimentin expression at the invasive front of tumor

To predict the prognostic value, survival analysis was carried by univariate Cox’s proportional hazard regression models in all the 127 patients with OSCC. The overall survival curve for the 127 samples was shown in Fig. 5a. The expression levels of CD68-positive macrophages (*P* = 0.009, Fig. 5b) and CD163-positive macrophages (*P* = 0.015, Fig. 5d) in tumor nest was significantly associated with poor prognosis. Furthermore, the expression levels of CD163 (Fig. 5g) in tumor cells (*P* = 0.001), vimentin both in tumor (*P* = 0.006, Fig. 5j) and at the tumor invasion front (*P* = 0.002, Fig. 5k) had a worse prognostic impact on the survival rate. However, the number of CD68-positive macrophages (Fig. 5c) and CD163-positive macrophages (Fig. 5e) in tumor stroma, and the levels of CD68 (Fig. 5f) and E-cadherin (Fig. 5h, 5i) expression in tumor cells did not shown any significant correlation with overall survival (Table 8).

**Table 8:** Univariate cox regression analysis of overall survival

covariate	*P*-value	Risk ratio	95 % CI
Sex (male, female)	0.4	1.35	(0.655, 2.043)
Age (≤50 or younger,>50)	0.13	2.49	(1.300, 3.680)
Tumor location Post1	0.75	1.16	(0.244, 2.082)
Tumor location Post2	0. 448	1.38	(0.551, 2.201)
Tumor location Post3	0.98	0	(−1242.048, 1242.048)
Tumor location Post4	0.99	0	(−1596.148, 1596.148)
Tumor location Post5	0.77	0.8	(−0.692, 2.288)
Tumor size (T1-T4)	0.45	1.18	(0.757, 1.595)
Nodal metastasis (N0, N(+))	0.44	1.31	(0.618, 2.006)
Metastasis (M0, M(+))	0.69	0.05	(0.760, 1.470)
Clinical stage (I, II, III, IV)	0.55	1.12	(1.146, 2.200)
Pathological grade	0.06	1.67	(0.244, 2.082)
CD68+ macrophages counts in tumor nest (Low, High)	0.01	3.08	(2.236, 3.914)
CD68+ macrophages counts in tumor stroma (Low, High)	0.3	0.69	(−0.010, 1.390)
CD163+ macrophages counts in tumor nest (Low, High)	0.02	2.83	(1.991, 3.669)
CD163+ macrophages counts in tumor stroma (Low, High)	0.48	0.78	(0.085, 1.473)
CD68 expression in tumor (Negative, Low, High)	0.97	1.01	(0.555, 1.465)
CD163 expression in tumor (Negative, Low, High)	0	2.2	(1.735, 2.667)
E-cadherin expression in tumor (Negative, Low, High)	0.61	0.89	(0.465, 1.323)
E-cadherin expression at the invasive front of tumor (Negative, Low, High)	0.19	0.74	(0.280, 1.1937)
Vimentin expression in tumor (Negative, Low, High)	0.01	1.79	(1.373, 2.207)
Vimentin expression at the invasive front of tumor (Negative, Low, High)	0	1.88	(1.474, 2.295)

Bold values signify *P* < 0.05

### The establishment and characterization of TAMs

**Fig. 6:**
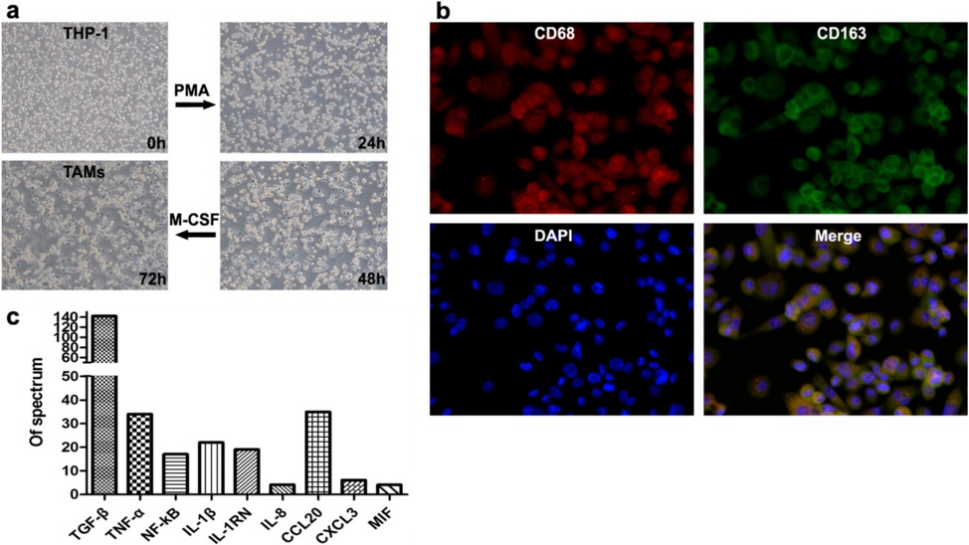
The establishment and characterization of tumor-associated macrophages. **a** The PMA-treated THP-1 cells were differentiated into adherent macrophages. **b** The Double immunofluorescence staining of CD68 (red) and CD163 (green) in the TAMs. The nuclei were stained with DAPI. The photographs were taken at 200x magnification. **c** The inflammatory cytokines and chemokines were detected in conditioned medium by using mass spectral analysis

Human THP-1 cells are always used as models to investigate the monocyte/macrophages differentiation [20]. In the current study, THP-1 cells quickly extended pseudopodia and became attached after the treatment with phorbolmyristate acetate (PMA, 10 ng/mL) for 48 h. Thereafter, the macrophage colony-stimulating factor (M-CSF) stimulates them to further differentiate into adherent macrophages (Fig. 6a). The expression of CD68 and CD163 on these macrophages was detected by immunofluorescence. As shown in Fig. 6b, these macrophages expressed not only CD68 (red) but also CD163 (green). Using mass spectral analysis, a variety of inflammatory cytokines and chemokines, including TGF-β, TNF, IL and so on (Fig. 6c) were present in the collected CM. Taken together, we confirmed that the TAMs were established and exhibited anti-inflammatory (M2) phenotype [20].

### TAMs induce the cancer cells undergo EMT in oral squamous cell carcinoma

**Fig. 7:**
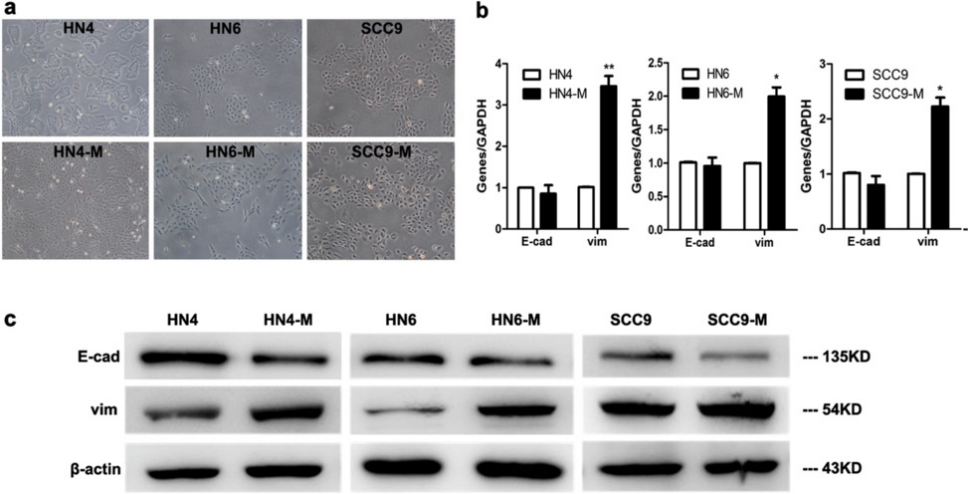
TAMs induce the cancer cells undergo epithelial to mesenchymal transition (EMT). **a** The HN4, HN6 and SCC9 cells presented typical cobblestone and epithelial-like appearances. The HN4-M, HN6-M and SCC9-M cells were scattered and spindle shaped, exhibited fibroblast-like appearances. The photographs were taken at 40x magnification. **b** The relative mRNA expression levels of E-cadherin, and vimentin was performed by real-time RT-PCR. The housekeeping gene GAPDH was used as the control. The data were reported as mean ± SD. **c** The EMT-related proteins E-cadherin and vimentin were examined by the Western blot, β-actin was used as a loading control. The data were reported as mean ± SD. * *P* < 0.05, ** *P* < 0.01

**Fig. 8:**
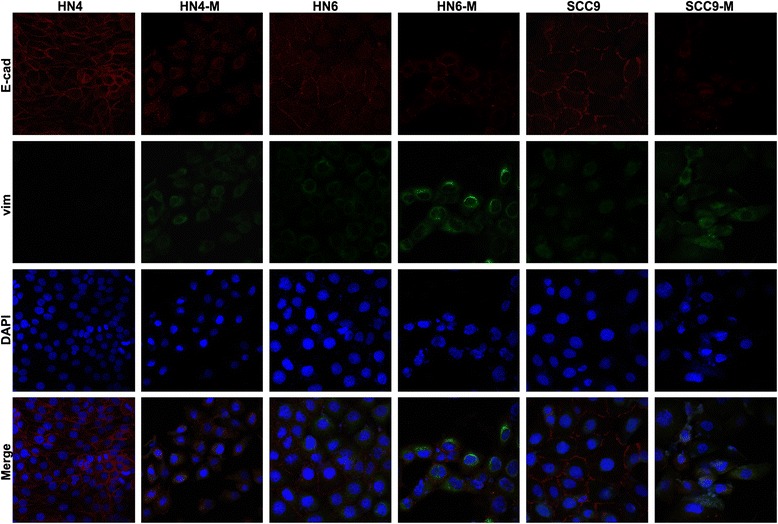
The Double immunofluorescence staining of E-cadherin (red) and vimentin (green) in the cells. The nuclei were stained with DAPI. The HN4-M, HN6-M and SCC9-M cells were scattered, exhibited fibroblast-like appearances, showed increased expression of vimentin and decreased expression of E-cadherin (400x magnification)

To clarity whether TAMs could induce the cells underwent EMT in OSCC, we treated the HN4, HN6 and SCC9 cells with the CM from TAMs and the morphology was observed. HN4-M, HN6-M and SCC9-M cells lose tight cellular junctions and became scattered and spindle shaped, appearing like fibroblasts (Fig. 7a). To further determine the EMT, the pattern of gene expression was analyzed by real-time PCR. The expression of E-cadherin decreased slightly, while vimentin expression increased sharply in HN4-M (3.46-fold, *P* < 0.05), HN6-M (1.99-fold, *P* < 0.05), and SCC9-M (2.22-fold, *P* < 0.05) cells (Fig. 7b). In line with the gene expression, Western blot analysis showed that the HN4-M, HN6-M and SCC9-M cells exhibited a significant decrease of E-cadherin, but an increase of the mesenchymal marker vimentin (Fig. 7c). In addition, we used double-staining immunofluorescence analysis to investigate the pattern of E-cadherin and vimentin. As Fig. 8 shown, the HN4-M, HN6-M and SCC9-M cells showed typical fibroblast-like and spindle-shaped appearance, with reduced E-cadherin but enhanced vimentin. Taken together, these results indicated that the TAMs could induce EMT in OSCC.

### TAMs enhance the cancer cells migration and invasion in vitro

**Fig. 9:**
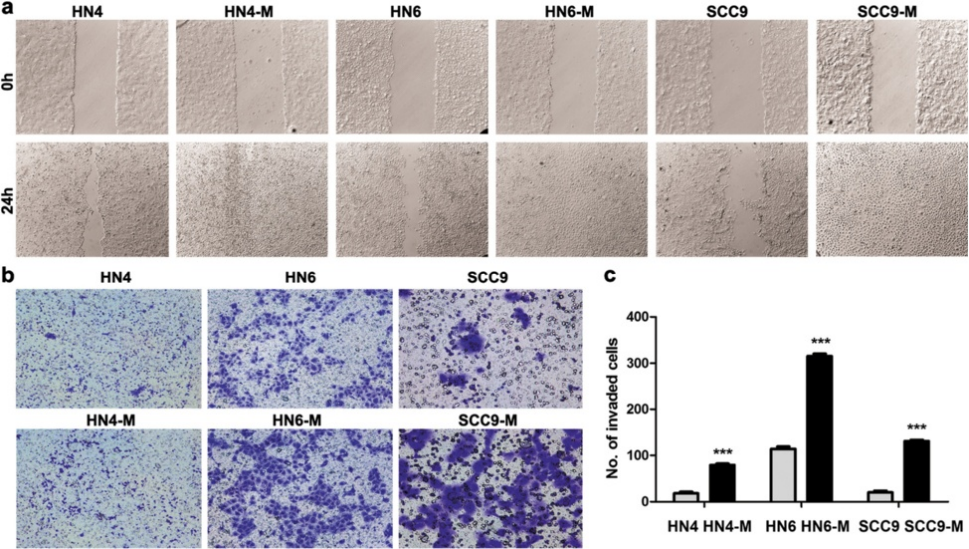
TAMs enhance the cancer cells migration and invasion in vitro. **a** The HN4-M, HN6-M and SCC9-M cells could grow to confluency easier than the control cells after 24 h. **b** The cells invading the filters coated with the Matrigel. The photographs were taken at 100x magnification. (**c**) The quantification of the cell invasion in five randomly chosen fields. The data were reported as mean ± SD. *** *P* < 0.001

To verify the migration of cancer cells, a wound-healing assay was used. The CM promotes the cancer cells to migrate and the wound was closed easily after 24 h as compared to untreated cancer cells (Fig. 9a). Furthermore, Transwell assays were carried to measure the invasive ability of the cells. After 24 h, the invasive cells were quantified and captured with × 100 magnification in five randomly chosen fields (Fig. 9b). After the treatment of CM, the cancer cells showed an increase in invasion with HN4-M (4.34-fold, *P* < 0.001), HN6-M (2.76-fold, *P* < 0.001) and SCC9-M (6.55-fold, *P* < 0.001) cells (Fig. 9c). These results demonstrated that the TAMs could induce EMT and enhance the migration and invasion of OSCC.

## Discussion

Increasing studies have shown that TAMs play a significant role in tumor progression and correlate with poor prognosis in numerous cancers. However, the interaction between the TAMs and clinicopathological features and the induction of EMT in OSCC has not been elucidated clearly. Exploration of the underlying mechanisms may reveal new targets for the therapy of oral cancers.

In the OSCC specimens, we found that the high number of CD68-positive macrophages in tumor nest was positively associated with tumor location and mortality. Using univariate cox analysis also revealed that the high number of CD68-positive macrophages was correlated with a poor overall survival. Meanwhile, the tumor stroma was also infiltrated by CD68-positive macrophages. However, a significant correlation between the high number of CD68-positive macrophages and the clinicopathological parameters and overall survival was not shown in stroma. These results were in accordance with the previous studies in solid malignancies that CD68 was frequently used as a marker to identify TAMs, and the dense infiltration of CD68-positive macrophages in the tumor was associated with poor survival [21, 22]. Previous studies have confirmed the TAMs include the M2 phenotype and CD163 is regarded as a specific marker of M2 macrophages. The present study showed the density of CD163-positive macrophages both in tumor nest and stroma significantly correlated with the overall survival. Our results support the previous researches that the infiltration of CD163-positive macrophages associated with poor prognosis in various tumors, such as hepatocellular carcinoma, renal cell carcinoma and bladder cancer [11, 23, 24]. Of note, the CD163-positive macrophages may be more suitable to identify the TAMs than the CD68-positive macrophages in OSCC and the infiltration of CD163-positive macrophages in tumor may be more important for tumor progression [8, 10, 11].

It has been reported that both CD68 and CD163 express on circulating monocytes and macrophages and used as TAMs markers in tumor progression [25, 26]. The present study demonstrated that CD68 and CD163 not only express in TAMs, but also in cancer cells. Interestingly, the expression of CD68 in tumor cells was only significantly associated with lymph node metastasis, but not with recurrence and survival. Although few previous studies have shown that the CD68 expressed in tumor cells, our novel findings was supported by Strojnik and coworkers, whose researches confirmed that the cancer cells exhibit expression of CD68 and the high CD68 staining of cancer cells correlated with poor prognosis [27]. Furthermore, CD163 was also present in tumor samples. Importantly, the expression of CD163 in cancer cells was significantly associated with recurrence and mortality. The univariate cox analysis also shows that the high expression of CD163 in cancer cells has a significant prognostic impact on overall survival. Based on our knowledge, this is the first study that demonstrated the expression of CD163 in cancer cells and its correlation with poor prognosis in OSCC, which is consistent with the studies in breast cancer, colorectal cancer and malignant melanoma [6, 9, 28, 29]. Macrophage antigens CD163 expressed in tumor cells may be a heterotypic cell fusion between the TAMs and tumor cells and this fusion may enhance the metastatic potential of tumor cells [12]. Another explanation is the cancer cells may be reprogrammed by the TAMs and transdifferentiate to a more mesenchymal-like property that may be more invasive and metastatic [12, 30]. Taken together, we hypothesized that the TAMs are closely correlated with EMT and may be important for cancer invasion and metastasis. This hypothesis was warranted for the further investigation.

To investigate whether the TAMs are involved in EMT, we detected the expression of E-cadherin and vimentin in OSCC patient samples. In contrast to our previous studies, the absence or reduced expression of E-cadherin was not significantly associated with the clinical variables [13]. The reason for the contradictory results may be due to the small sample size. However, the increased expression of vimentin in OSCC was significantly associated with lymph node metastasis, clinical stage, pathological grade, recurrence and overall survival. Also our data suggest the expression of CD163 in tumor cells was significantly correlated with the expression of E-cadherin and vimentin. Such novel results are supported by a previous study that CD163 expressing tumor cells may establish a subpopulation of tumor cells, which could exhibit an EMT-correlated phenotypic shift and increased metastatic ability induced by TAMs [31]. This may extend the explanation for the contradictory conclusions of E-cadherin in the present study. However, in order to investigate the interaction between TAMs and cancer cells with EMT in OSCC, we further established the TAMs in vitro and collected the CM from TAMs for the followed study. The incubation of TAMs CM resulted in a fibroblast-like appearance of cancer cells (HN4, HN6 and SCC9) together with the decreased/increased expression of E-cadherin/vimentin, which consistent with the findings suggest that TAMs are related with EMT transdifferentiation program in cancer cells by showing that downregulation of E-cadherin expression and acquisition of mesenchymal characteristics [31–33]. It has been reported that the cancer cells undergoing EMT was always accompanied by an increasing in cell motility and metastasis [14, 15]. Our results demonstrated that HN4-M, HN6-M and SCC9-M cells could migrate into the scratched area easily, the cells become more motile and correlated with metastasis. Furthermore, the cells underwent EMT also acquired high invasive ability. These findings, together with the analysis of clinical samples, provide an extend explanation that the TAMs could induce the EMT of cancer cells, which loss epithelial feature, present mesenchymal phenotype and acquire increase invasion and metastatic properties.

## Conclusions

In conclusion, this is the novel study shows macrophage traits and clinical significance in OSCC. The expression of CD68 and CD163 were not only confined to the infiltrating TAMs, but also presented in cancer cells. Furthermore, the expression of CD163 both in macrophages and in cancer cells is associated with poor overall survival and has a significant prognostic impact in oral cancer. Importantly, the cancer cells express CD163 has a significant relationship with E-cadherin and vimentin expression, which indicate TAMs are involved in the EMT, and further studies are essential to elucidate that the TAMs could induce the oral cancer cells undergo EMT and show highly invasive and metastatic behavior. Our study underlines the importance of the mutual interaction between the TAMs and cancer cells undergoing EMT in tumor progression.

## Abbreviations

CM: conditioned medium
EMT: epithelial to mesenchymal transition
OSCC: oral squamous cell carcinoma
TAMs: Tumor-associated macrophages
